# Radicle pruning by seed‐eating animals helps oak seedlings absorb more soil nutrient

**DOI:** 10.1111/1749-4877.12489

**Published:** 2020-10-10

**Authors:** Xianfeng YI, Minghui WANG, Chao XUE, Mengyao JU

**Affiliations:** ^1^ College of Life Sciences Qufu Normal University Qufu China

**Keywords:** ^15^N labeling, biomass allocation, oak, radicle pruning, seedling development

## Abstract

Although radicle pruning has well been observed in plant–animal interactions, research has not been conducted to determine how radicle pruning by seed‐eating animals regulates nutrition mobilization of cotyledonary reserves and absorption of soil nutrients. We used stable nitrogen isotopes to test how acorns of early‐germinating oak species (*Quercus variabilis*, *Q. aliena*, and *Q. mogolica*) trade off nutrients in the cotyledons and those in the soil in response to radicle pruning by seed‐eating rodents. Radicle pruning by rodents resulted in root branching in the 3 early‐germinating oak species. Moreover, radicle pruning increased shoot dry weight and substantially reduced the root‐to‐shoot ratio of oak species. Corresponding to the decreased dry weight of roots and root‐to‐shoot ratio, the dry weight of the remnant cotyledons was higher after radicle pruning in the 3 oak species. We provided first evidence that radicle pruning by seed‐eating animals improved seedling performance of early‐germinating oaks by increasing absorption of nutrients from soil. The results indicate that early‐germinating oak seedlings trade off nutrition budget by altering nutrient absorption from soil and reserve mobilization from cotyledons in response to radicle pruning by seed‐eating animals. Our study provided new insight into the nutrition allocation mechanism of young seedlings in response to radicle pruning by seed‐eating animals, reflecting a mutualistic interaction between early‐germinating oak and food‐hoarding animals.

## Graphical abstract

Although radicle pruning has well been observed in plant–animal interactions, research has not been conducted to determine how radicle pruning by seed‐eating animals regulates nutrition mobilization of cotyledonary reserves and absorption of soil nutrients. We used stable nitrogen isotopes to show evidence that radicle pruning by seed‐eating animals improved seedling performance of early‐germinating oaks by increasing absorption of nutrients from soil, providing new insight into the nutrition allocation mechanism of young seedlings in response to radicle pruning by seed‐eating animals, reflecting a mutualistic interaction between early‐germinating oak and food‐hoarding animals.

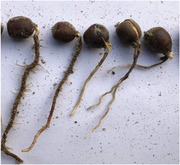

## INTRODUCTION

With a few exceptions, oaks can be generally grouped into 2 major categories, the white oak group and the red oak group (Fox 1982; Yi *et al*. 2012). Different from red oak species and other dormant plant species, early‐germinating oaks produce recalcitrant acorns that germinate shortly after seed fall in autumn due to little or no radicle dormancy (Smallwood *et al*. 2001; Tilki & Alptekin 2006; Ganatsas *et al*. 2017; Benamirouche *et al*. 2018). The rapid germination of acorns and development of taproots have well been considered an adaptive strategy of early‐germinating oak acorns to escape predation by seed‐eating animals (Fox 1982; Yi *et al*. 2012, 2013). Rapid germination of recalcitrant seeds has been suggested to influence seed selection and seed dispersal by seed‐eating animals (Hadj‐Chikh *et al*. 1996; Xiao *et al*. 2009) because nutrition reserves from cotyledons are rapidly transferred into the robust radicles after germination (Fox 1982).

Discrimination against seeds with high perishability has been presumably attributed to the short duration of food storage in caches made by hoarders (Hadj‐Chikh *et al*. 1996; Smallwood *et al*. 2001; Steele *et al*. 2001a,b). To maximize rewards from their caches, however, several squirrel species have been found to excise the embryo of white oak acorns to cease germination (Steele *et al*. 2001b, 2006; Xiao *et al*. 2010). Unlike squirrels, Siberian chipmunks *Tamias sibiricus* do not excise the embryo but only remove the protruding radicles of *Quercus mongolica* acorns before caching (Yang *et al*. 2012). This typical radicle pruning behavior has been widely found in the temperate and tropical rodents that clip off the distal end of the radicle of germinating seeds of recalcitrant plants before caching (Jansen *et al*. 2006; Cao *et al*. 2011; Zhang *et al*. 2016, 2017a). Radicle pruning slows down seed germination (Yang *et al*. 2012; Zhang *et al*. 2016), representing an alternative behavior of food hoarding animals to cope with rapid germination of white oak acorns.

Although radicle pruning may show negative impacts on seed germination (Yang *et al*. 2012), seedling establishment from radicle‐removed seeds appears not to be substantially affected (Yi & Liu 2014; Liu *et al*. 2016; Zhang *et al*. 2016). Despite failing to take the radicle‐pruning behavior of food‐hoarding animals into consideration, previous studies have shown that radicle pruning shows no effect on survival, shoot height, and diameter of seedlings, or shoot dry mass of *Q* *uercus* *vulcanica* and *Fagus orientalis* (Tilki & Alptekin 2006; Calikoglu *et al*. 2007; Devine *et al*. 2009). However, other studies indicate that radicle pruning of germinated acorns affects seedling morphology in several oak species (Schettler & Smith 1980; Bonner 1982; Gilman & Yeager 1987; McCreary 1996). A large body of literature has provided consistent evidence that radicle pruning promotes the formation of multiple taproots and increases root collar diameters and root surface area in various oak species (Harris *et al*. 1971; Barden & Bowersox 1989; McCreary 1996; Ertas 2002; Tilki & Alptekin 2006; Devine *et al*. 2009; Liu *et al*. 2016; Zhang *et al*. 2016).

It has been proposed that the multiple taproots originated from the trim points after radicle pruning will generate greater root surface area for the absorption of nutrients, and consequently promoting seedling growth (Tilki & Alptekin 2006). However, previous studies have provided evidence that cotyledonary reserves rather than soil nutrition play an important role in supporting development and growth of 1‐year oak seedlings (Mancilla‐Leytón *et al*. 2013; Yi & Liu 2014; Yi *et al*. 2014, 2015; Yi & Wang 2016; Shi *et al*. 2017, 2018). Therefore, we should not assume that multiple taproots induced by radicle pruning will necessarily result in enhanced absorption of soil nutrients. Moreover, existing studies mainly focused on the influence of radicle pruning on root system development and potential application for producing container seedlings with multiple root systems for a better quality transplant (Devine *et al*. 2009; Liu *et al*. 2016). To our best knowledge, research has not been conducted to determine how radicle pruning shortly after acorn germination regulates nutrition mobilization of cotyledonary reserves. In addition, little work has attempted to examine whether the formation of multiple taproots induced by radicle pruning increases absorption of soil nutrients and then promotes seedling development. Here, we used stable nitrogen isotopes to test how acorns of early‐germinating oak species (*Quercus variabilis*, *Q. aliena*, and *Q. mogolica*) trade off nutrients in the cotyledons and those in the soil in response to radicle pruning by seed‐eating rodents. If we accept that radicle pruning promotes the formation of multiple taproots, increases in root collar diameters, and enlargement of root surface area in oak species, it can be expected that (i) more nutrients will be absorbed from soil after radicle pruning; (ii) less reserves will be mobilized from acorn cotyledons due to radicle pruning; and (iii) radicle pruning will be beneficial to seedling development of white oaks as measured by seedling performance.

## MATERIALS AND METHODS

### Oak species

In September 2014, we collected acorns of cork oak *Q. variabilis* and Chinese white oak *Q. aliena* in the Tianchishan mountains, Henan province (central China). Acorns of Mongolian oak *Q. mongolica* were collected in the Xiaoxing'anling mountains, Heilongjiang province (northeast China). We collected acorns of each species from at least 10 individual trees to make a composite sample. All acorns were stored at 4 °C until the germination experiment. Acorns of these oak species have been reported to be subjected to radicle pruning by several seed‐eating rodents (Yang *et al*. 2012; Zhang *et al*. 2016, 2017a; Deng *et al*. 2020).

### Radicle pruning

In September 2014, we placed sound acorns of each oak species in a tray of moist vermiculite at 20 °C to allow germination in Dailing, Heilongjiang province. After the radicle had protruded from each acorn and reached the same length for proper comparison (around 2 cm), the acorn was randomly assigned to the pruned or control group to test the effect of radicle pruning on seedling establishment from early‐germinating oak acorns. In the radicle pruning group, the germinating acorns of each oak species were presented to individuals of Siberian chipmunks *T. sibiricus* caged in the artificial enclosures measuring 10 m × 10 m × 2.5 m in the morning, allowing them to clip down the radicles from acorns when caching them. We then collected the radicle‐pruned acorns from caches of *T. sibiricus* in the afternoon. In total, 32 radicle‐pruned and 32 control acorns with protruding radicles were randomly selected for later germination (hereafter shoot emergence) for each oak species. Acorns were then individually replanted 2 cm in depth in 15‐cm containers and placed under standard conditions in Nanchang of Jiangxi province, allowing further shoot emergence. Shoot emergence was checked every day for 20 days and then at irregular intervals of 2–3 days for another 20 days.

### Nitrogen isotope labeling

To determine how radicle pruning affects the distribution of soil nutrients into seedlings, ^15^N‐labeled fertilizer was supplied as 10 mmol⋅L^−1^ NH_4_Cl enriched to 98 atom% ^15^N (Shanghai Laiang Biotech Co., Ltd., China) on the same day when acorns were planted. Each container received 200 mL ^15^N‐labeled fertilizer (approximately 28 mg ^15^N). Containers were kept at room temperature under 600–800 μmol⋅m^−2^⋅s^−1^ radiation of fluorescent lamps, regularly watered, and randomly arranged in space until December 2014.

### Seedling growth measurement

To see the effect of radicle pruning on biomass allocation, each seedling was carefully dug out and cleaned under running water 60 days after sowing when the first flush finished (all leaves were fully developed). After counting the number of taproots of each seedling, they were oven‐dried (70 °C for 48 h) and then the dry masses of remnant cotyledons, roots, and shoots of each seedling were weighed separately to the nearest ±0.01 g. Root‐to‐shoot mass ratio was calculated using dry weight of shoot and root of seedlings individually.

### Allocation of soil‐derived nitrogen in oak seedlings

Dry shoots of five randomly selected seedlings germinated from the radicle‐pruned and control groups were separately ground for each oak species, respectively. Then, the samples were sent to the Laboratory of Stable Isotope Spectrometer, Chinese Academy of Forestry Sciences (Beijing), for analyses of N stable isotope abundances. We used a mixing isotope model to determine the proportion of soil‐derived nitrogen allocated into oak seedlings according to the method used by Yi and Wang (2016) as follows:

$$\begin{array}{ccc} N\%= & & \left({}^{15}N_{\mathrm{seedling}}{-}^{15}N_{\mathrm{cotyledons}}\right)/\\ & & \left({}^{15}N_{\mathrm{fertilizer}}{-}^{15}N_{\mathrm{cotyledons}}\right)\times 100 \end{array}$$

where N%, ^15^N_seedling_, ^15^N_cotyledons_, and ^15^N_fertilizer_ stand for the soil‐derived N allocated into seedlings, the ^15^N isotope abundance of shoots, natural acorns, and ^15^N fertilizer (98 atom%), respectively. A previous study provides evidence that some N from the fertilizer can be translocated to the cotyledons during ^15^N labeling, resulting in very low ^15^N enrichment compared to the plant organs and the fertilizer (Villarsalvador *et al*. 2010). Therefore, we used the mean ^15^N isotope abundance of the cotyledons prior to isotope labeling, which was estimated as 0.477% for the oak species used in this study (Yi & Wang 2016).

### Statistical analysis

We performed data analyses using the SPSS 16.0 Package. Repeated measures were used to test if radicle pruning affects acorn germination. Chi‐square test was used to test how radicle pruning influenced the final seedling establishment. A two‐way ANOVA was used to determine the effects of radicle pruning and oak species on the dry masses of shoots, roots, as well as remnant cotyledons, followed by a post hoc test such as the least significant difference method for multiple comparison. The same procedure was applied to test if radicle pruning and oak species affect the number of taproots, root‐to‐shoot ratio, and the proportion of soil‐derived N allocated into the oak seedlings.

## RESULTS

**Figure 1:**
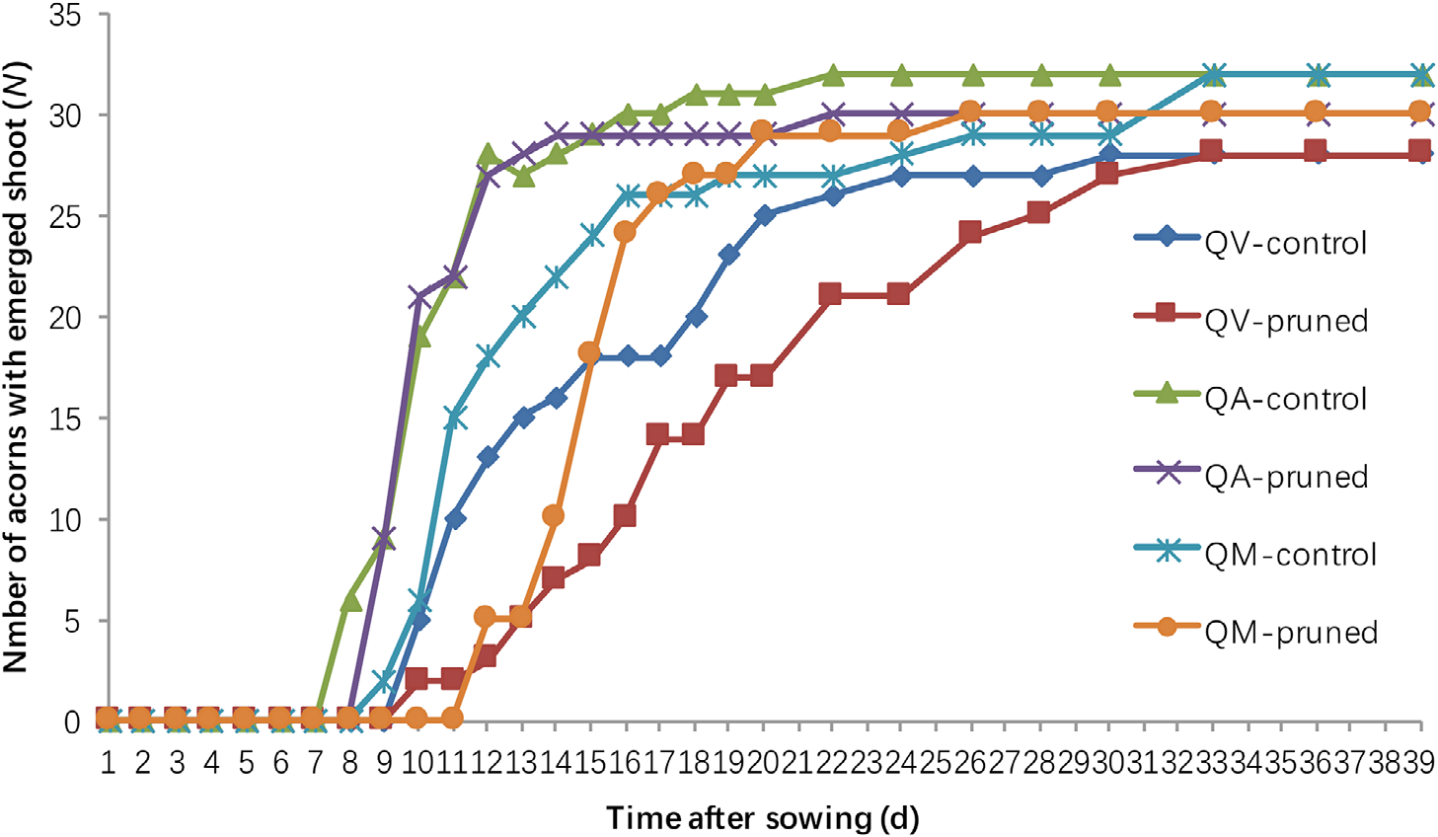
Effects of radicle pruning by chipmunks on shoot emergence of *Quercus variabilis* (QV), *Q. aliena* (QA), and *Q. mongolica* (QM) acorns.

Siberian chipmunks ubiquitously pruned the radicles before caching acorns. Repeated measures analysis revealed that radicle pruning by the chipmunks did not significantly slow down acorn germination of either oak species (*Wald* = 0.305, df = 1, *P* = 0.581; *Wald* = 0.000, df = 1, *P* = 1.000; *Wald* = 1.563, df = 1, *P* = 0.211). Chi‐square test showed that the final seedling establishment was not affected by radicle pruning (χ^2^ = 0.533, df = 5, *P* = 0.991) (Fig. 1).

**Figure 2:**
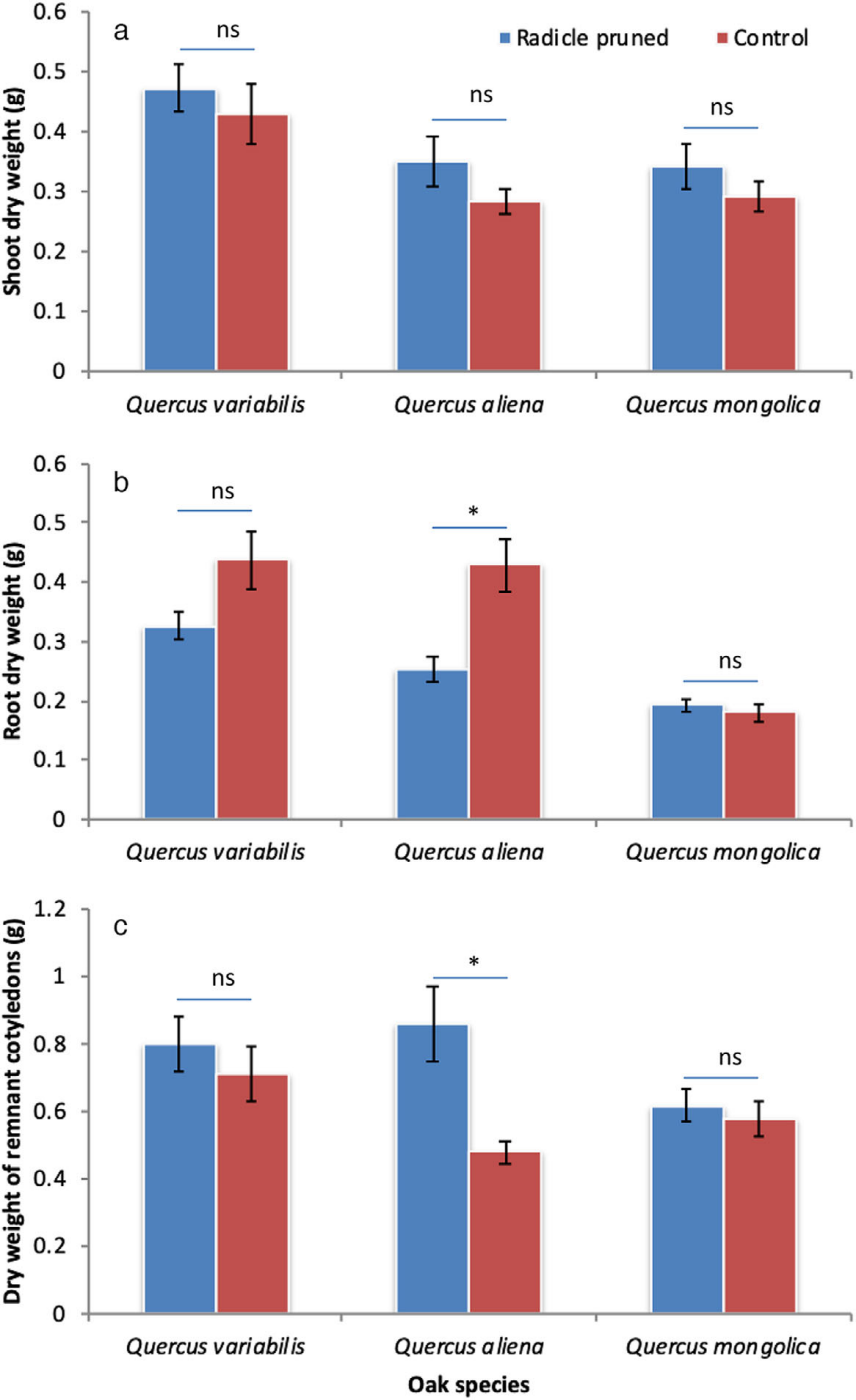
Effects of radicle pruning by chipmunks on dry weight of shoots (a), roots (b), and remnant cotyledons (c) of seedlings of *Quercus variabilis* (QV), *Q. aliena* (QA), and *Q. mongolica* (QM). Data are expressed as mean ± SD. Asterisks on the histogram indicates statistically significant at *P* = 0.05 level, while ns means not statistically significant.

**Figure 3:**
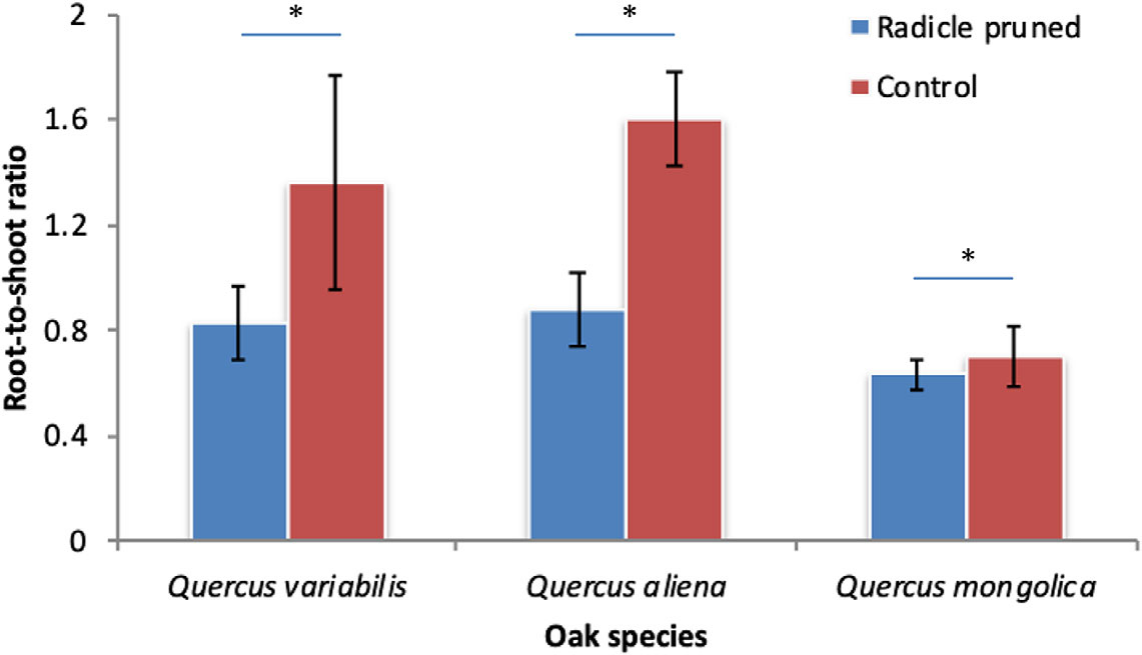
Effects of radicle pruning by chipmunks on root‐to‐shoot ratio of seedlings of *Quercus variabilis* (QV), *Q. aliena* (QA), and *Q. mongolica* (QM). Data are expressed as mean ± SD. Asterisks on the histogram indicates statistically significant at *P* = 0.05 level, while ns means not statistically significant.

Two‐way ANOVA indicated that early‐germinating oak species increased in shoot dry weight when radicles were pruned by the chipmunks compared with their uncut controls (*Q. variabilis*: 0.47 vs 0.43 g; *Q. aliena*: 0.35 vs 0.28 g; and *Q. mongolica*: 0.34 vs 0.29 g; *F* = 4.994, df = 2, *P* = 0.029) (Fig. 2a). Post hoc test showed that shoot dry weight of *Q. variabilis* was much higher than those of *Q. aliena* and *Q. mongolica* (*F* = 2.705, df = 2, *P* = 0.076). However, radicle pruning by the chipmunks significantly decreased the root dry weight (*Q. variabilis*: 0.33 vs 0.44 g; *Q. aliena*: 0.25 vs 0.43 g; and *Q. mongolica*: 0.19 vs 0.18 g; *F* = 7.189, df = 1, *P* = 0.010) regardless of oak species (Fig. 2b). Post hoc test showed that *Q. variabilis* and *Q. aliena* were much higher in root dry weight than *Q. mongolica* (*F* = 13.329, df = 2, *P* < 0.001) (Fig. 2b). The dry weight of remnant cotyledons was higher when radicles were pruned compared with the control group (*Q. variabilis*: 0.80 vs 0.72 g; *Q. aliena*: 0.86 vs 0.48 g; and *Q. mongolica*: 0.62 vs 0.58 g; *F* = 5.280, df = 1, *P* = 0.025), but remained similar among the 3 oak species (*F* = 1.690, df = 2, *P* = 0.194) (Fig. 2c). Root‐to‐shoot mass ratio also decreased in a manner similar to root dry weight (*Q. variabilis*: 0.83 vs 1.36; *Q. aliena*: 0.88 vs 1.60; and *Q. mongolica*: 0.63 vs 0.71; *F* = 7.404, df = 1, *P* = 0.009). Post hoc test showed that seedlings of *Q. variabilis* and *Q. aliena* exhibited higher root‐to‐shoot ratio than those of *Q. mongolica* (*F* = 3.540, df = 2, *P* = 0.036) (Fig. 3).

**Figure 4:**
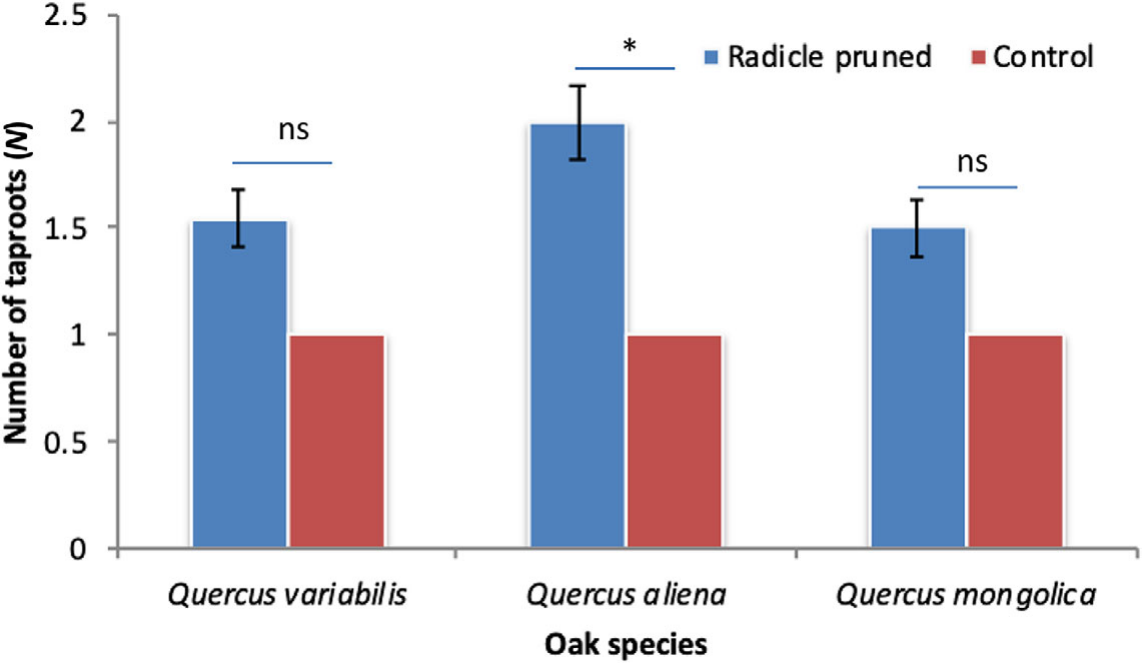
Effects of radicle pruning by chipmunks on the number of taproots of seedlings of *Quercus variabilis* (QV), *Q. aliena* (QA), and *Q. mongolica* (QM). Data are expressed as mean ± SD. Asterisks on the histogram indicates statistically significant at *P* = 0.05 level, while ns means not statistically significant.

**Figure 5:**
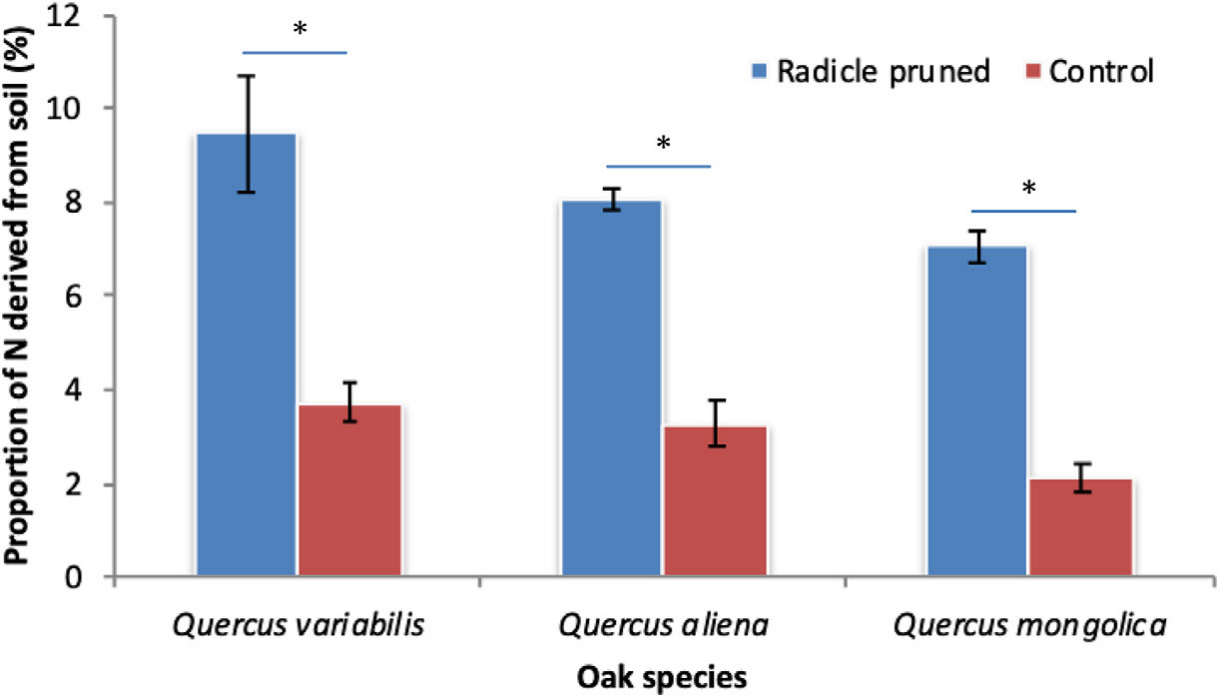
Effects of radicle pruning by chipmunks on soil nitrogen absorption of seedlings of *Quercus variabilis* (QV), *Q. aliena* (QA), and *Q. mongolica* (QM). Data are expressed as mean ± SD. Asterisks on the histogram indicates statistically significant at *P* = 0.05 level, while ns means not statistically significant.

Radicle pruning by chipmunks induced more multiple taproots compared with the control (*Q. variabilis*: 1.5 vs 1.0; *Q. aliena*: 2.0 vs 1.0; and *Q. mongolica*: 1.5 vs 1.0; *F* = 43.642, df = 1, *P* < 0.001). No effect of oak species on the number of taproots was detected both in the control and pruned acorns (*F* = 2.347, df = 2, *P* = 0.105) (Fig. 4). Oak seedlings re‐germinated from radicle‐pruned acorns absorbed more nitrogen from soil compared to the control (*Q. variabilis*: 9.45% vs 3.72%; *Q. aliena*: 8.09% vs 3.28%; and *Q. mongolica*: 7.06% vs 2.13%; *F* = 521.980, df = 1, *P* < 0.001). Consequently, seedlings of *Q. variabilis* and *Q. aliena* absorbed more nitrogen from soil compared to those of *Q. mongolica* (*F* = 26.086, df = 2, *P* < 0.001) (Fig. 5).

## DISCUSSION

In summary, radicle pruning by seed‐eating animals in our study showed no significant effect on acorn re‐germination and the final seedling establishment regardless of oak species, supporting the previous study (Zhang *et al*. 2016). Consistent with previous studies (Schettler & Smith 1980; Bonner 1982; McCreary 1996; Tilki & Alptekin 2006; Devine *et al*. 2009; Zhang *et al*. 2015), radicle pruning by *T. sibiricus* resulted in root branching in the 3 early‐germinating oak species. Consequently, we found that seedlings of the 3 oak species absorbed much more nitrogen from soil when the radicles were pruned.

These observations are not consistent with a previous study showing that radicle pruning fails to stimulate the formation of lateral roots (Sosa‐Rodriguez *et al*. 2014). Atzmon *et al*. (1994) showed that taproot pruning caused an immediate increase in the radioactive carbon accumulation in the upper lateral roots. Therefore, radicle pruning by seed‐eating animals appears to inhibit the development of a robust taproot, and then induce the formation of multiple taproots of germinating acorns. The decrease in root mass further indicates the production of a more branched fibrous root system, which is supposed to be beneficial to nutrient absorption (Tilki & Alptekin 2006). Moreover, radicle pruning by *T. sibiricus* increased shoot mass and substantially reduced the root‐to‐shoot mass ratio especially in 2 temperate oak species, *Q. variabilis* and *Q. aliena*, reflecting a reduction in the size of taproot favoring a more branched root system. The branched multiple taproots are expected to promote seedling development because root surface area will be larger than that of seedlings with a single taproot (Devine *et al*. 2009; Rodríguez *et al*. 2015). Therefore, it can be anticipated that seedlings producing a more branched root system due to radicle pruning by seed‐eating animals might be more successful in the field (Schultz & Thompson 1990; Liu *et al*. 2016; Van Sambeek *et al*. 2016).

Until now, the effect of radicle pruning on oak seedling performance seems to be controversial. Previous study showed that radicle pruning failed to adversely affect seedling production of Shumard (*Q. shumardii*) and cherrybark oak (*Q. falcata*) (Bonner 1982). Recently, however, Caliskan (2014) witnessed a positive effect of radicle pruning on seedling performances of holm oak (*Quercus ilex*) in terms of larger taproot production and increasing height and root collar diameter of seedlings. In the present study, radicle pruning significantly increased the root‐to‐shoot ratio and the number of taproots irrespective of oak species. These results are different from the study by Zhang *et al*. (2016) that radicle pruning by acorn‐eating animals shows no effect on seedling performance of *Q. variabilis* seedlings, but are in agreement with Mucha *et al*. (2018) showing that root‐pruning generates lower root mass and higher stem mass of seedlings. This difference can be largely attributed to the length at which the radicle is pruned or to the oak species investigated. For example, radicles pruned at 10 and 7 cm increase seedling performance of cherrybark oak when compared with the control (Bonner 1982), while radicles pruned to 0.5 cm show no improvement of holm oak seedlings when compared with the control (Caliskan 2014). Devine *et al*. (2009) further show that seedling shoot weight, root weight, and stem diameter of Oregon white oak (*Q. garryana*) are not significantly affected by radicle pruning 1 cm from the acorn. Similarly, shoot growth of other oak species has shown little response to radicle pruning (Barden & Bowersox 1989; McCreary 1996).

Corresponding to the decreased root mass and root‐to‐shoot ratio, radicle pruning by *T. sibiricus* tended to increase the mass of the remnant cotyledons of the three oak species. Change in the mass of remnant cotyledons during development of oak seedlings has been largely neglected in previous studies (Liu *et al*. 2016; Zhang *et al*. 2016). With the radicle being pruned by *T. sibiricus*, nutrients in the cotyledons are prevented from being transferred into the branched taproots, consequently producing small size of root system. Although cotyledonary reserves contributed less to seedling development when the radicle was pruned, shoot mass became larger in response to radicle pruning in the 3 early‐germinating oak species. This can be explained by the fact that oak seedlings absorbed much more nitrogen from soil when the radicle was pruned (Fig. 5). A more branched root system induced by radicle pruning may confer an advantage to oak seedlings, by producing a larger surface area of root system to absorb soil nutrients (Tilki & Alptekin 2006). Previous studies provide evidence that acorns of white oak species are important as a food reward for the seed dispersing animals, rather than supplying energy for the development of young seedlings (Bartlow *et al*. 2018; Yi *et al*. 2019). Therefore, early‐germinating oak seedlings trade off cotyledon reserve mobilization for increase nutrient absorption from soil in response to radicle pruning by seed‐eating animals. Although soil nutrients contribute little to seedlings compared to the cotyledonary reserves (Mancilla‐Leytón *et al*. 2013; Yi *et al*. 2015; Yi & Wang 2016; Shi *et al*. 2017, 2018), the results may show first evidence that radicle pruning by seed‐eating animals improves seedling performance of early‐germinating oaks by increasing root surface area for the absorption of nutrients from soil.

Although radicle pruning by seed‐eating animals is unable to prevent acorn germination (Yang *et al*. 2012; Zhang *et al*. 2016), the remnant cotyledons of oak seedlings usually contain a substantial proportion of nutrition (Yi *et al*. 2013; Yi & Wang 2016). Rodents and jays find and excavate cotyledons attached to seedlings by searching for emerging epicotyls of 1‐year seedlings (Bossema 1979; Yi *et al*. 2013; Zhang *et al*. 2017b). If we accept the notion that radicle pruning by seed‐eating animals both improves performance of seedlings and decreases mobilization of cotyledonary reserves, the behavior of radicle pruning may represent an adaptive strategy of food hoarding animals. Both oaks and food hoarding animals are expected to benefit from radicle pruning, reflecting a mutualistic interaction between early‐germinating oak and food hoarding animals.

## CONFLICT OF INTEREST

All authors declare no conflict of interest.

## AUTHOR CONTRIBUTIONS

X.Y. conceived and designed the study. M.W., C.X., and M.J. carried out the experiments. M.W. performed data analyses. X.Y. wrote the first draft of the manuscript, and all authors contributed substantially to revisions.

## Data Availability

Data will be deposited at Dryad Digital Repository upon acceptance.
